# Phylogeny of locusts and grasshoppers reveals complex evolution of density-dependent phenotypic plasticity

**DOI:** 10.1038/s41598-017-07105-y

**Published:** 2017-07-26

**Authors:** Hojun Song, Bert Foquet, Ricardo Mariño-Pérez, Derek A. Woller

**Affiliations:** Department of Entomology, Texas A&M University, College Station, TX USA

## Abstract

Locusts are grasshoppers that can form dense migrating swarms through an extreme form of density-dependent phenotypic plasticity, known as locust phase polyphenism. We present a comprehensive phylogeny of the genus *Schistocerca*, which contains both non-swarming grasshoppers and swarming locusts. We find that the desert locust, *S. gregaria*, which is the only Old World representative of the genus, is the earliest diverging lineage. This suggests that the common ancestor of *Schistocerca* must have been a swarming locust that crossed the Atlantic Ocean from Africa to America approximately 6 million years ago, giving rise to the current diversity in the New World. This also implies that density-dependent phenotypic plasticity is an ancestral trait for the genus. Through ancestral character reconstruction of reaction norms, we show that colour plasticity has been largely retained in most species in the genus, but behavioural plasticity was lost and regained at least twice. Furthermore, we show that swarming species do not form a monophyletic group and non-swarming species that are closely related to locusts often express locust-like plastic reaction norms. Thus, we conclude that individual reaction norms have followed different evolutionary trajectories, which have led to the evolutionary transition between grasshoppers and locusts - and vice versa.

## Introduction

Locusts are grasshoppers belonging to the family Acrididae (Insecta: Orthoptera) that can form dense migrating swarms through an extreme form of density-dependent phenotypic plasticity, in which cryptically coloured, shy individuals can transform into conspicuously coloured, gregarious individuals in response to increases in population density^1–3^. In addition to colour and behavioural changes, these insects exhibit density-dependent morphological, reproductive, developmental, physiological, biochemical, molecular, and ecological changes^3–7^. In nature, depending on local population density, locusts are polyphenic along the continuum between two extreme phenotypes, known as solitarious phase and gregarious phase^1, 3^. This syndrome of coordinated changes is known as locust phase polyphenism^1, 3, 8^.

The two phases that can result from locust phase polyphenism are “among the most striking coordinated alternative phenotypes known”^9^, and, in fact, locusts are now considered to be a model system for studying phenotypic plasticity^8, 10^. Phenotypic plasticity is often regarded as an adaptation to heterogeneous environments^11–13^ and a large body of literature has been dedicated to understanding the evolution and maintenance of adaptive phenotypic plasticity^9, 13–20^. Several phase-related traits in locusts have been shown to be adaptive^8^. For example, density-dependent colour change has been shown to be an effective anti-predatory strategy in the early stage of swarm formation, especially when it is coupled with preferential feeding on toxic plants^21–23^. Recent theoretical studies have also shown that density-dependent behavioural change could have evolved as an adaptation to reduce risks of cannibalism among individuals within nymphal bands^24^. Life-history traits that change in response to changes in density, including maturation period, longevity, and reproductive potential, are also shown to be adaptations against rapidly changing environments^3, 8, 21, 25, 26^.

An effective way for studying the evolution of phenotypic plasticity is by taking a reaction norm perspective^17^. A reaction norm is a set of phenotypes that can be produced by an individual genotype when exposed to different environmental conditions^17, 27, 28^. Reaction norms can be either plastic or non-plastic^17, 29^ and they can be the objects of selection^16, 17, 27^ or subject to evolutionary processes, such as drift^15, 30^. For instance, among the phase-related traits in locusts, nymphal colouration can be considered a kind of a reaction norm^31, 32^. Many locust species have a plastic reaction norm in which nymphal colouration changes from green to a conspicuous colour (a combination or red, orange, or yellow and black patterns) when crowded^3, 33^ while closely related non-swarming species may have a non-plastic reaction norm in which nymphal colouration does not change regardless of rearing density^32, 33^. The same idea can be applied to other phase-related traits, with potentially different underlying mechanisms regulating each reaction norm. In other words, locust phase polyphenism is a collection of individual plastic reaction norms that all respond to changes in density^32^.

Most of the studies on the evolution of phenotypic plasticity deal with variations in reaction norms within species or within populations and there are only a handful of studies focusing on multiple species in a comparative framework^32, 34–42^. However, an explicitly phylogenetic study of phenotypic plasticity, that is, tracing the evolution of reaction norms based on a robust phylogenetic hypothesis, is extremely rare. This is because obtaining an accurate phylogenetic hypothesis and quantifying reaction norms of multiple species in a clade are both challenging tasks. A comparative quantification of phenotypic plasticity is especially difficult because the existence of plasticity has to be empirically demonstrated through explicit experiments^16^. Nevertheless, a phylogenetic investigation into the evolution of phenotypic plasticity can result in valuable insights^38, 40, 41, 43–46^ because reaction norms can be considered as characters that evolve by descent with modification. If a group of extant species expresses plastic reaction norms and if these species are shown to be monophyletic based on a phylogenetic analysis, it is possible to attribute plasticity to common ancestry, rather than novel adaptation in each species^43^. Therefore, a phylogenetic approach in the study of phenotypic plasticity can provide a more accurate understanding regarding the polarity of the evolution of reaction norms^32, 38, 43^.

**Figure 1:**
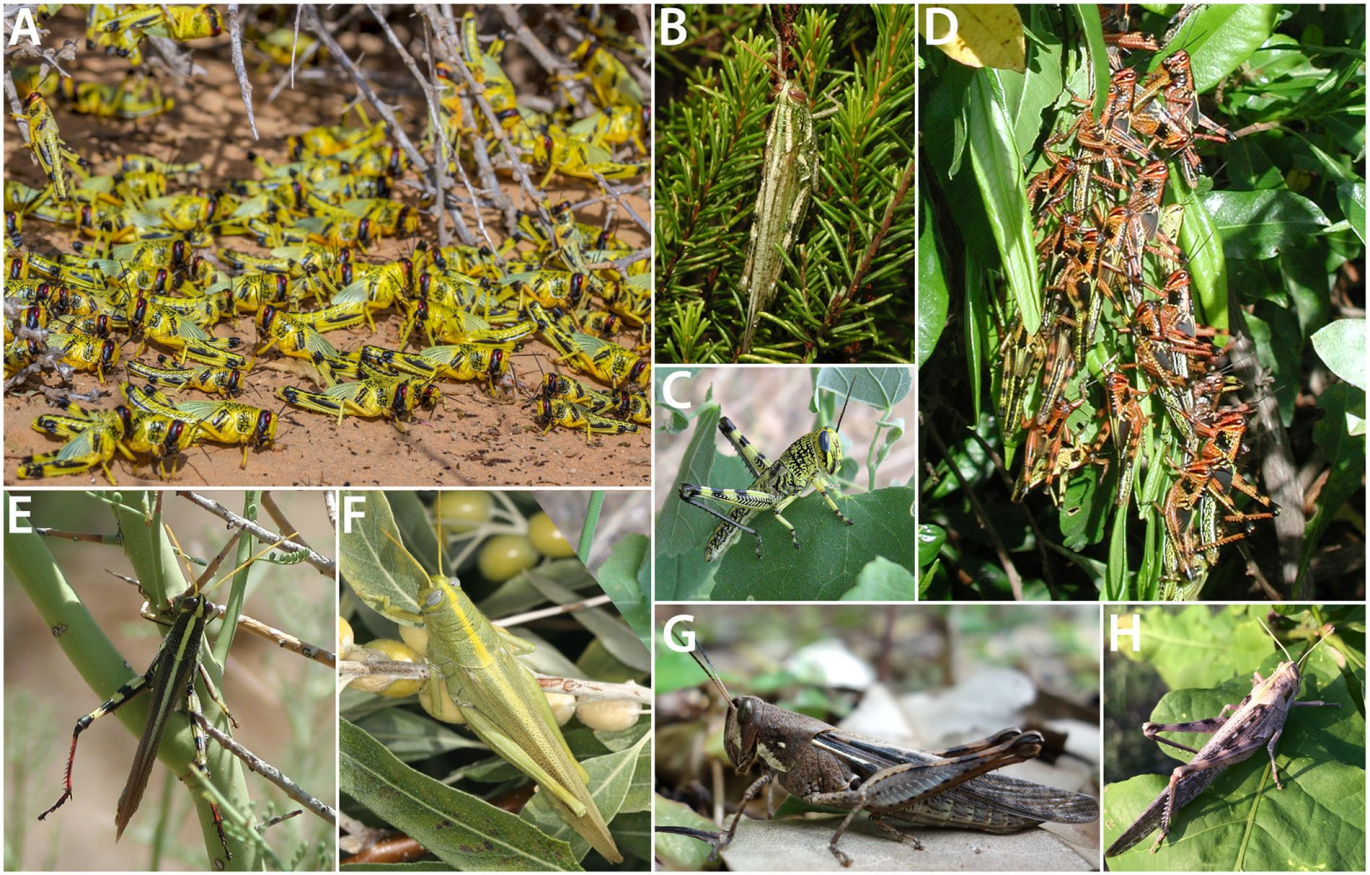
Some representatives of the genus *Schistocerca*; (**A**) Gregarious nymphs of *S. gregaria*; (**B**) *S. ceratiola*; (**C**) *S. lineata* nymph feeding on *Ptelea*; (**D**) Gregarious nymphs of *S. piceifrons*; (**E**) *S. albolineata*; (**F**) *S. shoshone*; (**G**) *S. caribbeana*; (**H**) *S. nitens* (Photo credit: Gil Wizen [**A**], Hojun Song [**B–H**]).

The genus *Schistocerca* Stål (Orthoptera: Acrididae: Cyrtacanthacridinae) is an excellent model system for studying the evolution of density-dependent phenotypic plasticity because it contains both locusts and non-swarming grasshoppers^32, 47^ (Fig. 1). Of about 50 species in the genus, only three species are considered to be true swarming locusts that have afflicted humanity over millennia by consuming agricultural yields en masse: the desert locust (*S. gregaria*), the Central American locust (*S. piceifrons*), and the South American locust (*S. cancellata*)^48^. There is a fourth species, *S. interrita*, which exhibited major outbreaks during 1983–1984 and 1997–2003 on the northern coast of Peru^49^, but this species is typically not known to swarm under normal conditions^33^ and not much is known about its biology. The remaining *Schistocerca* species are sedentary grasshoppers that do not swarm^32, 47^. Nevertheless, several sedentary species are known to express density-dependent phenotypic plasticity in colour, similar to the change expressed in the desert locust^32, 50–53^, while other species do not respond to changes in density at all^54, 55^. Furthermore, at least two sedentary *Schistocerca* species are known to change their behaviour in response to crowding^51^. In other words, a large amount of variation exists in the specific expression of density-dependent reaction norms within *Schistocerca*. Thus, by understanding the evolutionary relationships between locust species and non-swarming species that share a common ancestor, and by characterising density-dependent reaction norms across these species, it is possible to investigate the evolution of phenotypic plasticity in traits that collectively make up the complex syndrome of locust phase polyphenism.

The biogeography of *Schistocerca* provides a unique scenario for inferring whether density-dependent phenotypic plasticity is an ancestral or a derived trait for the genus. The subfamily Cyrtacanthacridinae, to which *Schistocerca* belongs, contains 35 genera and is distributed throughout the Old World, with most of its diversity in Africa^32^. Only two genera occur in the New World, *Halmenus*, a brachypterous genus endemic to the Galapagos Archipelagos^56^ and *Schistocerca*, which shows an unusual transatlantic distribution. It has been previously suggested that *Halmenus* is actually a brachypterous member of *Schistocerca* ^57, 58^. Within the genus, only the desert locust is found in the Old World (Africa and the Middle East) while all the other species occur in the New World (North, Central, and South America). To explain this interesting biogeographic pattern, two alternative hypotheses have been proposed^57, 59^. The desert locust was initially considered a migrant species from the New World^47^ because it is morphologically very similar to the species in the Americana Complex, which also includes other locust species in the genus^47, 48^, and because it is able to copulate with several New World species, although no viable offspring are produced^60^. This idea that an ancestral species originating from the New World colonised Africa and gave rise to the present-day desert locust is referred to as the New World Origin hypothesis. However, a highly unusual incident in 1988 prompted scientists to propose an alternative hypothesis. In October and November of 1988, large swarms of the desert locust successfully crossed the Atlantic Ocean from West Africa to reach the Caribbean and neighboring parts of South America^61–63^. This seemingly impossible flight was later postulated to have lasted only a few days, considering prevailing wind patterns (northeast trade winds) and the energy required to achieve the continuous flight of 5000 km^62, 64^. Based on this incident, Ritchie and Pedgley^63^ and Kevan^61^ proposed that the New World *Schistocerca* species were descendants of a “*gregaria*-like” ancestor from Africa that crossed the Atlantic Ocean. This second idea is referred to as the Old World Origin hypothesis.

These two hypotheses can be easily tested by the placement of *S. gregaria* in the phylogeny of *Schistocerca*. The New World Origin hypothesis can be supported if the desert locust is phylogenetically nested deep within the New World species. This would also indicate that density-dependent phenotypic plasticity is a derived trait within the genus. On the other hand, the Old World Origin hypothesis can be supported if the desert locust is placed basally to the rest of the New World *Schistocerca*, which would also mean that density-dependent phenotypic plasticity is an ancestral trait for the genus. Interestingly, both hypotheses have been previously supported based on different lines of evidence^57–59^, and the conflict has not been fully resolved.

Therefore, in this study, we reconstruct a robust phylogeny of *Schistocerca* to date using molecular data in order to better understand how locusts and non-swarming grasshoppers are related to each other and to test how colour and behavioural reaction norms have evolved in this genus. We demonstrate that the evolution of density-dependent phenotypic plasticity can be effectively studied in a phylogenetic framework and that different reaction norms of locust phase polyphenism show different patterns of divergence. Finally, we use the phylogeny and biogeography to understand the patterns of diversification in *Schistocerca* and how they relate to the evolution of density-dependent phenotypic plasticity.

## Results

### Phylogeny and biogeography of *Schistocerca*

**Figure 2:**
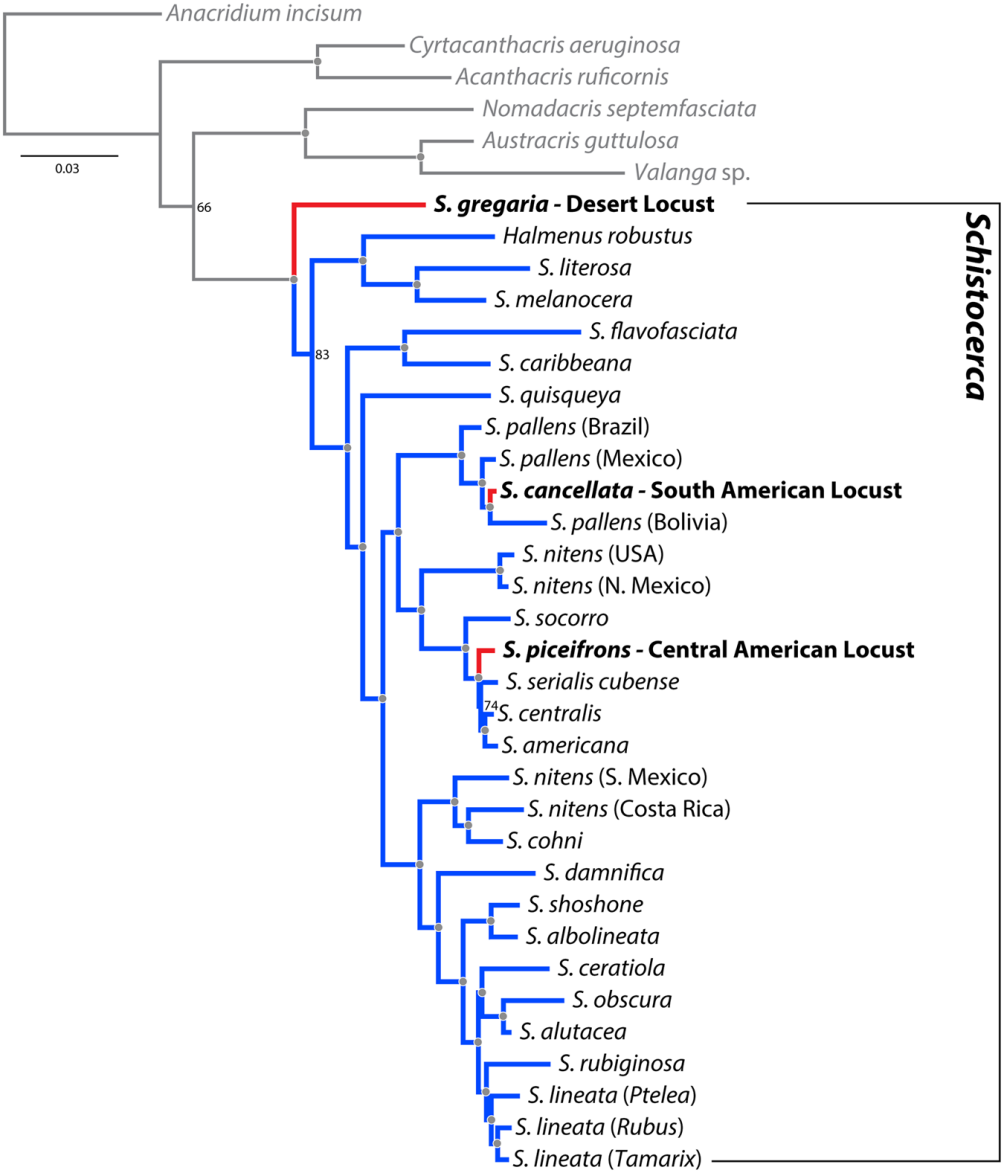
Bayesian tree of *Schistocerca* based on molecular data. Gray dots on the nodes denote posterior probability of 100. Nodal support values lower than 100 are specified. Red branches indicate swarming locusts and blue branches indicate sedentary grasshoppers.

**Figure 3:**
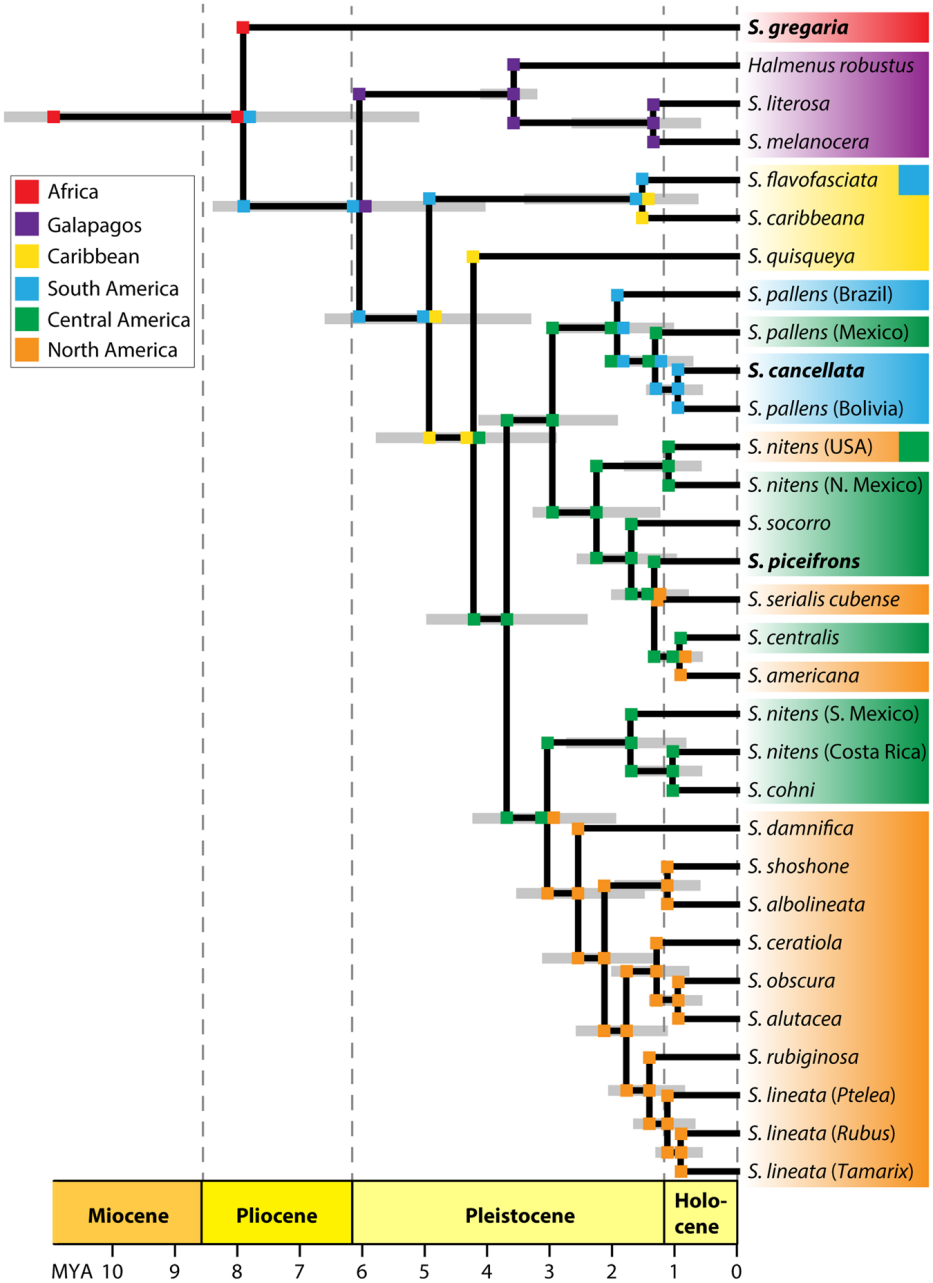
Time-calibrated tree of *Schistocerca* with estimated ancestral ranges inferred using DIVALIKE model implemented in BioGEOBEARS (d = 4.4589; e = 0.0287; LnL = −63.73). Outgroups are not shown. The species in bold face indicate swarming locusts. Both *S. flavofasciata* and *S. nitens* (USA) have broad distribution across two defined regions.

We used both maximum likelihood and Bayesian analyses to infer the phylogeny of *Schistocerca*, which resulted in identical topologies and recovered *Schistocerca* as a strong monophyletic group (Fig. 2). The divergence time analysis estimated that the genus originated about 7.9 million years ago (Fig. 3). Within the genus, the desert locust (*S. gregaria*), the only African representative, was placed at the base of the phylogeny, and the remaining New World *Schistocerca* species formed a monophyletic group. The colonisation of the New World by the ancestral *Schistocerca* from Africa was estimated to have taken place about 6 million years ago. Using the DIVALIKE model in BioGeoBEARS, we inferred that the ancestral *Schistocerca* colonised and diversified in South America first, and then went through radiation in Central and North America. The flightless genus *Halmenus*, represented by *H. robustus*, was the most closely related to *S. literosa* and *S. melanocera*, suggesting a single colonisation event to the Galápagos Islands by the common ancestor of the three species, which was estimated to have taken place 3.6 million years ago, soon after the islands became available for colonisation. The remaining New World species consisted of several clades that were geographically clustered, including the Caribbean, South America, North America, and Central America. Overall, we inferred the diversification of major lineages to have taken place during the Pliocene and continued throughout the early Pleistocene.

We found the previously proposed species groups to be largely paraphyletic. The Americana Complex *sensu* Harvey^48^ originally included *S. americana*, *S. piceifrons*, *S. cancellata*, *S. gregaria*, *S. serialis*, and *S. pallens*, but we found that these six species did not form a clade. The Alutacea Group *sensu* Song^65^, which included *S. alutacea*, *S. rubiginosa*, *S. lineata*, *S. shoshone*, *S. albolineata*, and *S. obscura*, was also paraphyletic due to the placement of *S. ceratiola*, which was found to be more closely related to *S. obscura* and *S. alutacea*. We included three specimens of *S. pallens* collected from Mexico, Brazil, and Bolivia, but they did not form a monophyletic group because the Bolivian specimen was sister to *S. cancellata*. Likewise, we included four specimens of *S. nitens* collected from U.S.A., northern and southern Mexico, and Costa Rica, but they also did not form a monophyletic group. Conversely, three specimens of *S. lineata*, a species known to feed on different host plants^66^, were also included and found to be monophyletic.

### Evolution of density-dependent phenotypic plasticity

**Figure 4:**
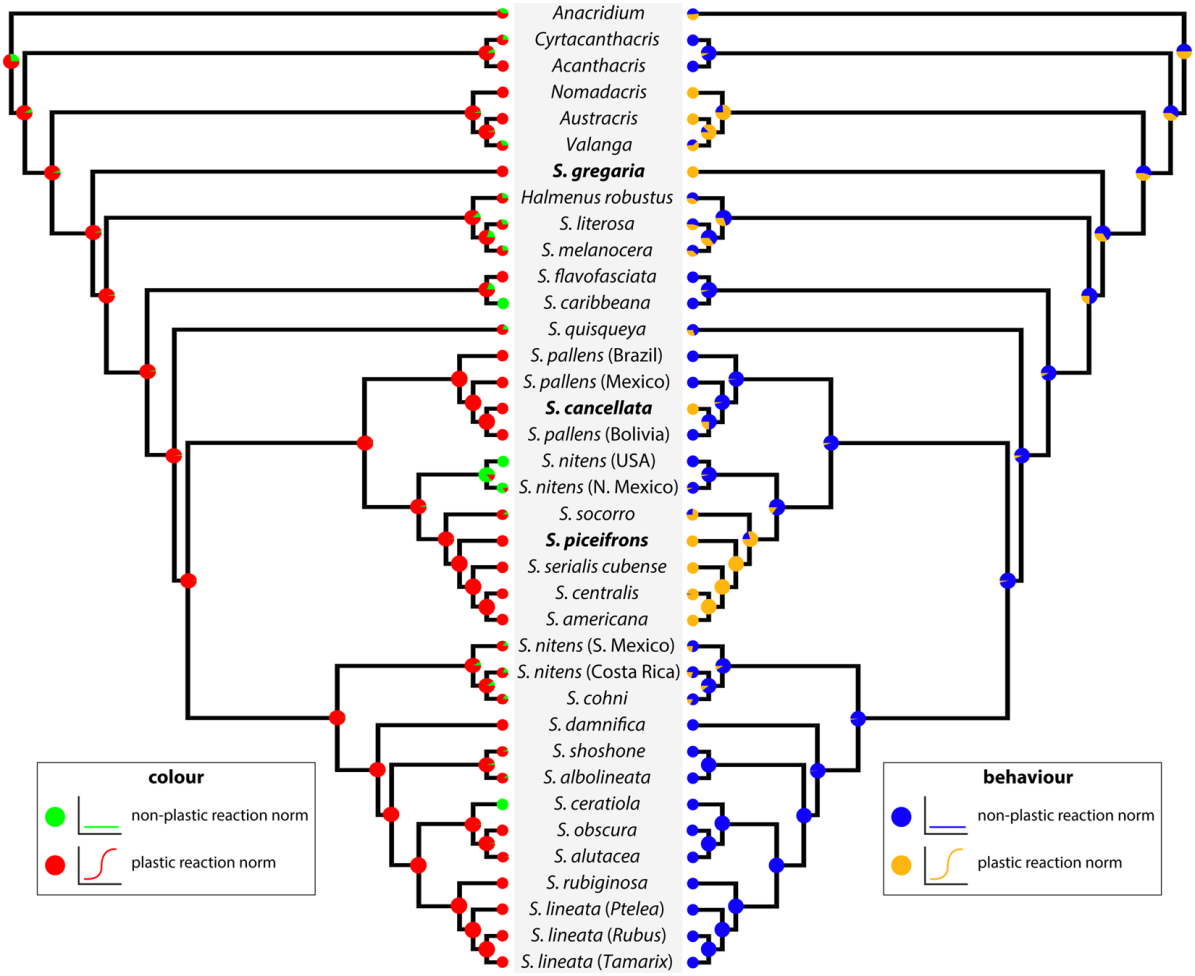
Ancestral character state reconstruction using stochastic character mapping. The left tree shows the evolution of density-dependent reaction norm in nymphal colouration and the right tree shows the evolution of density-dependent reaction norm in behaviour. The pie chart-like symbols on the nodes represent the probability of ancestral character state. The species in bold face indicate swarming locusts.

We found that the three locust species (*S. gregaria*, *S. piceifrons*, and *S. cancellata*) did not form a monophyletic group, suggesting that swarming locusts have evolved multiple times within *Schistocerca* (Fig. 2). We inferred that the ancestral *Schistocerca* species must have been a swarming locust due to the basal placement of *S. gregaria*, and that swarming behaviour was lost and regained at least twice: once in *S. piceifrons* and once in *S. cancellata*. The ancestral character reconstruction (Fig. 4) revealed that nearly all *Schistocerca* species, as well as the cyrtacanthacridine outgroups, express a density-dependent plastic reaction norm in nymphal colouration, which suggests that this could be a symplesiomorphic trait for *Schistocerca*. This plastic reaction norm appears to have been maintained throughout the diversification of the genus, but was independently lost at least three times: in *S. caribbeana*, *S. nitens* from the USA, and *S. ceratiola*. In contrast, we found the expression of a plastic behavioural reaction norm to be rare within the genus. Although *S. gregaria* shows a density-dependent plastic reaction norm in behaviour, which implies that the common ancestor of *Schistocerca* must have exhibited behavioural plasticity, it was quickly lost and the capacity to respond to density was not maintained at all throughout the diversification of the genus. Since the initial loss, this plastic reaction norm was regained twice within the genus. We also tested whether these two reaction norms have evolved in a correlated fashion using Pagel’s method^67^, and found that there was no correlation between them (Mk model, likelihood-ratio = −9.160668, *p*-value = 1). This pattern collectively suggests that two density-dependent plastic reaction norms that are often associated with locust phase polyphenism had very different evolutionary trajectories throughout the diversification of *Schistocerca*.

## Discussion

Phenotypic plasticity is rarely studied in a phylogenetic framework because generating a reliable phylogeny is not a trivial matter and it is also often difficult to quantify reaction norms of the taxa used in the analysis^16^. However, when such an approach is taken, it can provide unique insights into understanding the evolution of reaction norms^38, 40, 43^. In this study, we present the most definitive phylogeny of *Schistocerca* to date, which provides a comparative framework for elucidating how density-dependent phenotypic plasticity might have evolved in this clade (Fig. 2). We have also reconstructed ancestral character states of density-dependent reaction norms based on this phylogeny to demonstrate that the two reaction norms that are most associated with locust phase polyphenism, colour and behaviour, have followed two very different evolutionary trajectories throughout the diversification of the genus (Fig. 4).

It has long been recognised that understanding the evolutionary relationships between locusts and non-swarming grasshoppers within *Schistocerca* is the key to unraveling the evolution of locust phase polyphenism^57–59^. In particular, the phylogenetic position of the desert locust (*S. gregaria*) relative to the rest of the genus has major implications for inferring the polarity of reaction norm evolution. According to our phylogeny, the desert locust is the earliest diverging lineage within the genus, implying that the ancestral *Schistocerca* must have originated from Africa and colonised the New World, thereby supporting the Old World Origin hypothesis. The controversy regarding the origin of the desert locust was previously examined from a phylogenetic perspective based on two different lines of evidence. Song^59^ proposed the first phylogeny of *Schistocerca* using 57 morphological characters, which placed the desert locust deep within the Americana Complex mostly based on the shape of pronotum, male cerci, and male genitalia. This phylogenetic position of the desert locust supported the New World Origin hypothesis, but the study was unable to adequately demonstrate how the ancestral *Schistocerca* might have colonized the New World in the first place. Song^59^ invoked a potential transpacific colonization by the ancestral *Schistocerca* from the Indo-Pacific to South America based on the recovered topology of cyrtacanthacridine outgroups, but provided little evidence for this assertion. Lovejoy *et al*.^57^ presented the first molecular phylogeny of *Schistocerca* based on 1.7 kb fragments of mitochondrial genome spanning ND1 to 12S, which robustly placed the desert locust at the base of *Schistocerca* phylogeny, supporting the Old World Origin hypothesis. Their phylogeny is highly congruent with our phylogeny, which contains nearly 10 times the amount of sequence data. The discrepancies in phylogenetic signal between morphological and molecular data deserve some explanations. While it is now clear that the desert locust is the earliest diverging lineage within the genus, it is morphologically most similar to the swarming species in the Americana Complex, which do not share the most recent common ancestor with the desert locust. This pattern could suggest that there might have been a strong selective pressure for different swarming locust species to converge on a similar body form, so much so that most of the taxonomically important characters are actually homoplasious. Also, because *Schistocerca* is a relatively recently-derived genus, it is possible that not enough morphological differences have yet accumulated among species. In fact, compared to other grasshopper groups, the male genitalia, which are typically the most taxonomically useful characters in grasshoppers, are essentially identical across the entire genus^47, 65^. Thus, we consider that Song’s^59^ morphological phylogeny was heavily affected by convergent characters, which might have led to incorrect biogeographic inferences.

We estimate that the westward colonisation from Africa by the ancestral *Schistocerca* took place during the Pliocene, when the continents were essentially identical to their current configuration. Thus, the westward transatlantic flight from Africa to America is the only plausible explanation for the current distribution^57, 62, 64^. When the swarms of the desert locust crossed the Atlantic Ocean in 1988, they all perished without establishing any viable populations^62^, but it is not too far-fetched to imagine that such transatlantic dispersals could have happened in the past, and one of these events might have resulted in the original stock for the New World *Schistocerca*. A recent study^68^ based on a paleoclimate simulation proposed that the aridification of the Sahara Desert was caused by the shrinkage of the Tethys Sea during the Tortonian state (7–11 million years ago) of the Late Miocene, coinciding with the estimated origin of *Schistocerca* (Fig. 3). This aridification process could have created favourable environmental conditions for the ancestral *Schistocerca* to evolve an extreme form of density-dependent phenotypic plasticity. Studies have also suggested that the aridification in subtropical West Africa coincided with the intensification of northeast trade winds^69^, which blew from northwestern Africa to the Caribbean islands and northern South America. If the swarms of the ancestral *Schistocerca* took advantage of the northeast trade winds for crossing the Atlantic Ocean, the place it arrived must have been a very different place from where they originated. During the Pliocene, South America essentially consisted of tropical forests and patches of savanna^70^. In this new environment, it would have been more adaptive for these locusts to be in the solitarious phase than to be swarming locusts, which could have eventually led to the loss of swarming behaviour in descendant species.

We have established that the presence of density-dependent phenotypic plasticity is an ancestral trait for the genus. When we dissect this phenotypic plasticity into smaller reaction norms, we begin to see two very different evolutionary patterns. One of these, nymphal colouration, is the most obvious reaction norm that responds to density in locusts^32^. In the desert locust, individuals reared in isolation are typically green, and those reared in crowded conditions develop black patterns with yellow background colour^1^ (Fig. 1A). Green nymphal colouration is cryptic and helps the locusts to blend into the vegetation^30^. Sword *et al*.^23^ showed that when gregarious nymphs of the desert locust in Mauritania feed on a native toxic plant, *Hyoscyamus muticus*, at the onset of population density increase, their contrasting colour pattern could function as a warning colouration. Thus, this plastic reaction norm might have evolved in the context of an anti-predator strategy^8, 22^. However, our survey of the effect on crowding in nymphal colouration across Cyrtacanthacridinae based on both literature and experimental data shows that this plastic reaction norm is not a novel trait for *Schistocerca*, but actually a conserved and widespread trait throughout the subfamily^32^. Although it is not clear in what context this plastic reaction norm in nymphal colouration evolved in the first place, the density-dependent aposematism shown in the desert locust appears to be a case of exaptation.

Because this plastic reaction norm is a symplesiomorphic trait for the genus, any *Schistocerca* species that changes colour in response to crowding can be inferred to have retained this reaction norm and any species that does not change colour when crowded can be inferred to have lost the plasticity. Although we had missing data for 16 taxa (43.2% missing), we were able to reconstruct the ancestral reaction norms and predict the probability of plastic reaction norms for the missing taxa using the stochastic character mapping approach^71^ (Fig. 4). We infer that the plastic reaction norm in nymphal colouration has largely persisted throughout the diversification of the genus. However, it is worth noting that the expression of this plastic reaction norm varies quite a bit amongst the New World *Schistocerca* species^32^. For example, the gregarious nymphs of the Central American locust (*S. piceifrons*) (Fig. 1D) and the South American locust (*S. cancellata*) express contrasting colouration patterns of red and black or yellow and black, respectively^33^. Two sedentary species closely related to the Central American locust (*S. americana* and *S. serialis cubense*) exhibit similar red and black patterns when experimentally crowded^51^. Also, a central Texas population of *S. lineata*, a sedentary species that feeds on a toxic plant (*Ptelea trifoliata*) exhibits density-dependent warning colouration^72^ and shows bright yellow and black patterns when crowded (Fig. 1C).

Most other sedentary *Schistocerca* species, however, do not undergo such a dramatic colour change, but rather a subdued one^32^, usually changing from green to light yellow, reddish brown, orange, or tan with black mottling patterns. These colour patterns are not consistent with aposematic colouration and it is also unknown whether these species feed on toxic plants when crowded. In nature, most of these sedentary species typically do not occur in high densities and nymphs are nearly always green. We think that these species have the genetic capacity to change colour in response to density mainly due to shared ancestry, but this plasticity is normally not expressed. We also think that it is probably not costly to maintain this variation, and there has not been a strong selective pressure against maintaining this plasticity. Interestingly, there are three species in the genus that exhibit a non-plastic reaction norm in nymphal colouration. Two of them, *S. caribbeana* and *S. nitens*, are polychromatic as nymphs, meaning that isolated nymphs are not only green, but also brown or gray^55^. Nymphs of the third species, *S. ceratiola*, are green with white stripes and black dots regardless of the rearing density. For these non-plastic species, there could have been strong selective pressure to completely lose the plasticity or a stochastic process, such as drift, might have led to fixation of a non-plastic reaction norm. What is possibly even more interesting is that these three species are phylogenetically divergent from each other, which suggests that the loss of plasticity has happened independently.

The ability to change behaviour in response to change in population density is at the core of what defines locusts^3, 8^. Solitarious locusts tend to be shy and inactive, and avoid other locusts, while gregarious locusts tend to be highly active and mobile, and attracted to each other^73^. In the desert locust, it only takes a few hours to induce behavioural gregarisation^74^ and it is known that a combination of visual and olfactory stimuli, or a tactile stimulus on hind femora alone, can induce change in behaviour^75^. Although the ancestral condition for *Schistocerca* was the expression of plastic reaction norm in behaviour, this condition does not seem to have persisted throughout the diversification of the genus, unlike the pattern we observe in nymphal colouration. The ancestral state reconstruction suggests that behavioural plasticity was quickly lost in the common ancestor of the New World *Schistocerca*, only to reappear twice (Fig. 4).

Recent theoretical and empirical studies have suggested that migrating locusts, especially as nymphal bands, are in a generally poor nutritional state^76^, and that the threat of intraspecific cannibalism can influence the evolution of local interactions and collective movement in the desert locust^77^. In other words, density-dependent behavioural plasticity could be considered an adaptation to reduce risks of cannibalism^24^. As alluded to earlier, the ancestral *Schistocerca* that colonised the New World probably experienced an environment that was nutritionally rich, assuming that the plants it encountered were edible. This means that the selective pressure to maintain the expression of plastic reaction norm in behaviour in northern Africa was no longer present, or it could have been even maladaptive to express behavioural plasticity in this new environment. Furthermore, the availability of resources may have been more abundant and less variable temporally in the New World habitats compared to the heterogeneous desert habitats, possibly leading to less variable population dynamics. These two factors could then have led to the loss of behavioural traits that were originally adapted to life in a crowd. Such an example can be found in the South African subspecies of the desert locust, *S. gregaria flaviventris*. This subspecies is geographically isolated from its nominal species by 2,500 km and occurs between the Kalahari Desert and the Western Cape of South Africa, characterised by Succulent Karoo and Fynbos^78–80^. This subspecies is genetically distinct from the nominal species and shows a much-reduced level of density-dependent phenotypic plasticity^79^. Although this subspecies still inhabits arid areas in South Africa, it has more access to a continuous supply of vegetation compared to its northern relative, which might have contributed to the loss of the plastic reaction norm in behaviour. In the New World, many of the sedentary *Schistocerca* species have evolved preferences for host plants and adopted a very different life history strategy from locusts^72, 81–83^. In this sense, it is actually surprising that the plastic reaction norm in behaviour was regained twice in *Schistocerca*. On the other hand, one of these species, the South American locust (*S. cancellata*), is adapted to arid or semi-arid regions in northern Argentina^78^ and it is possible that similar selective pressures that the desert locust experiences may have promoted the regaining of behavioural plasticity in this species. Conversely, the Central American locust (*S. piceifrons*) is a tropical locust^84^, which swarms in the presence of lush vegetation, and it is not yet clear what factors might have promoted the re-expression of plastic reaction norm in behaviour in this species. What is more interesting is that the sedentary species that are closely related to *S. piceifrons* actually express a reduced amount of density-dependent behavioral plasticity^51^. This indicates that reaction norm in behaviour expressed in *Schistocerca* is not necessarily binary (present/absent), but can evolve or be lost gradually.

Considering the different evolutionary trajectories of colour and behavioural plasticity in *Schistocerca*, it is important to consider the proximate mechanisms behind these reaction norms. Studies have shown that the black patterns associated with gregarious nymphs are due to a hormonal factor in the brain and corpora cardiaca, known as [His^7^]-corazonin^85^, which is a conserved peptide that has a darkening effect in many lineages within Orthoptera^86^. A recent study shows that the knockdown of corazonin (*Crz*) gene is known to induce lightening of body colour^87^, further confirming the role of this peptide. The corazonin-mediated colour change does not happen instantaneously and may take up to several instars, implying that the plastic reaction norm in nymphal colouration requires physiological machinery that is slow-acting. Our study suggests that this physiological machinery has been phylogenetically conserved throughout the diversification of *Schistocerca*. On the contrary, the plastic reaction norm in behaviour, at least in the desert locust, is a rapid response to the changes in density, mediated by a conserved neurotransmitter, serotonin^88, 89^. The change in behaviour can occur within hours because the gregarious behaviour is a response to a sudden pulse of serotonin in the metathoracic ganglion^90^. A direct application of serotonin on to the thoracic ganglia elicits behavioural gregarisation^88, 90^. It has also been shown that a cAMP-dependent protein kinase (PKA), which is a downstream effector of one of several serotonin receptors in insects (5HT_7_), as well as an intracellular effector molecule closely linked to learning, is also required for this transition^91^. However, long-term gregarious nymphs actually express low levels of serotonin^92^, meaning that its effect on behavioural gregarisation is transient. It also appears that it is not the overall increase of serotonin in the thoracic hemocoel^93^, but a sudden increase in the specific areas within thoracic ganglia that elicits behavioural gregarisation. A recent immunofluorescence study^89^ showed that the desert locust has three classes of serotonergic neurons in their thoracic ganglia that respond to either all gregarising stimuli, only sight and smell of other locusts, or long-term crowding or isolation. So far, the mechanism of serotonin production and the effect of serotonin as related to locust phase polyphenism has only been examined in the desert locust^89, 90, 92^, but it is possible to postulate that a similar pattern might be found in other swarming *Schistocerca* species, but not in sedentary species. Thus, within *Schistocerca*, what might have evolved in locust species could be the ability to quickly produce a high level of serotonin in thoracic ganglia in response to the increase in density. However, it should be noted that there are other biogenic amines that are involved in behavioural gregarisation^90, 92^ and that serotonin is not ubiquitously important in all locust species. For example, in the migratory locust (*Locusta migratoria*), dopamine plays a more important role in modulating phase changes^94, 95^, while serotonin enhances solitarious behaviour^96^.

**Figure 5:**
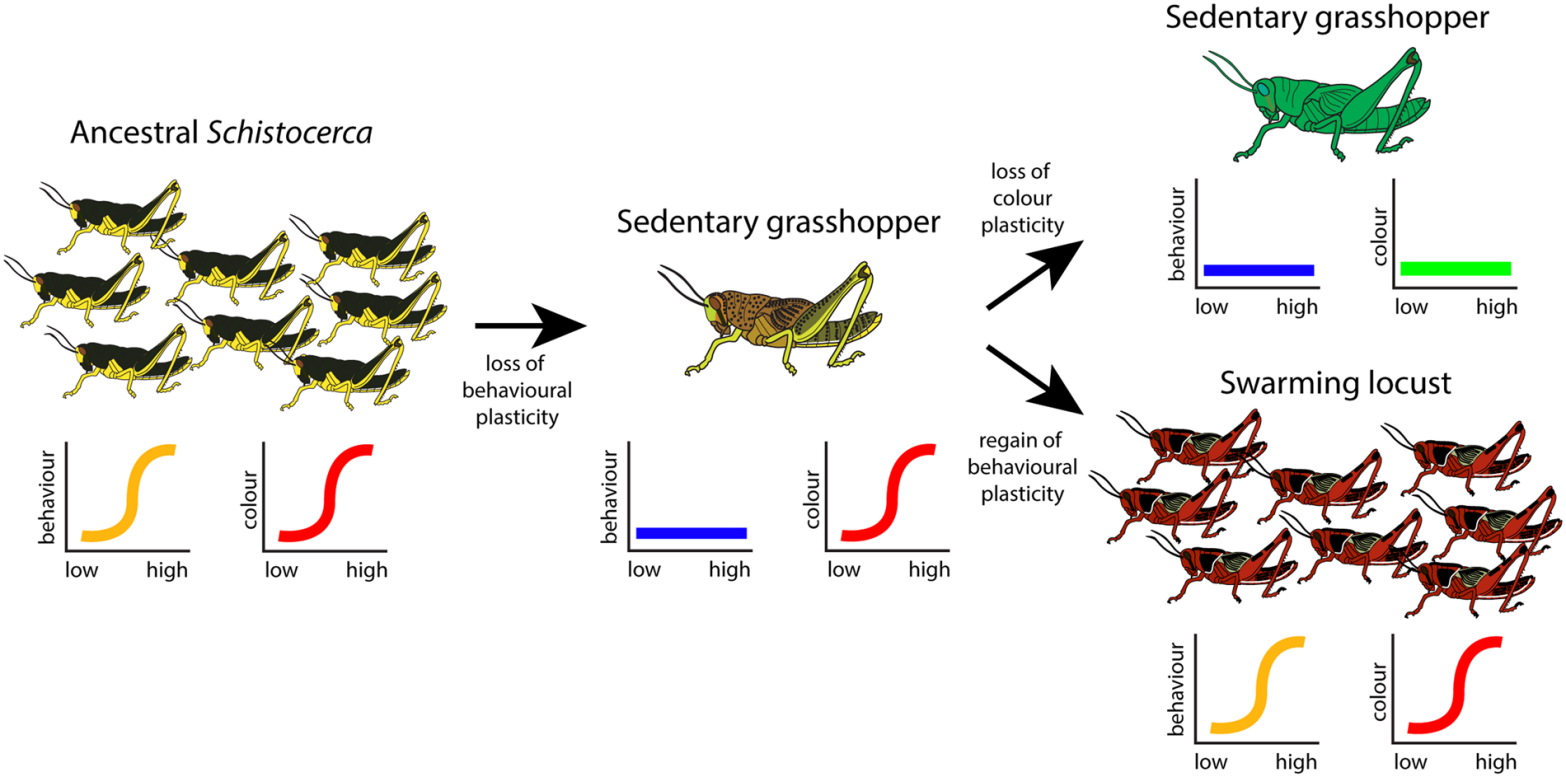
A graphical summary of evolution of density-dependent reaction norms as inferred from this study. The ancestral *Schistocerca* was a swarming locust and must have had plastic reaction norms in both behaviour and colour. However, behavioural plasticity was lost in the descendant species while colour plasticity was maintained. In some species, colour plasticity was lost and they have become completely non-responsive to density. There has been at least two independent re-gains of behaviour plasticity giving rise to two locust species in the New World.

So far, much of what we know about the mechanism of locust phase polyphenism has come from the study of the desert locust^3, 8^. In this study, we have established a robust phylogenetic framework and the polarity of the evolution of reaction norms. We have also shown that the plastic reaction norms in colour and behaviour have evolved separately and that there is no evidence that they have evolved in a correlated fashion (Fig. 5). Based on these findings, we can now begin to examine specific physiological, neurological, and molecular mechanisms of density-dependent phenotypic plasticity in the Central American locust and the South American locust, as well as other non-swarming sedentary species, to compare with the mechanisms already established in the desert locust. In particular, it would be of great interest to test whether the proximate mechanism of behavioural gregarisation, which must have been lost and regained within *Schistocerca*, is similar or different between the desert locust and the other locust species. Finally, we conclude that the phylogeny of *Schistocerca* has enabled new insights into the complex evolution of phenotypic plasticity and has opened a door to understand the back-and-forth evolutionary transitions between grasshoppers and locusts.

## Methods

### Taxon and character sampling

We sampled a total of 37 taxa, including 6 cyrtacanthacridine genera as outgroups and 31 ingroup taxa representing the genus *Schistocerca*. Because previous molecular studies have consistently suggested that the Galapagos endemic genus *Halmenus* is, in fact, flightless *Schistocerca* ^57, 58^, we included *H. robustus* as one of the ingroup taxa. We also included multiple samples of three widely distributed species (*S. pallens*, *S. nitens*, and *S. lineata*) collected from different geographic localities to test their monophyly. Our ingroup sampling consisted of three major locust species (*S. gregaria*, *S. piceifrons*, and *S. cancellata*) as well as phylogenetically diverse lineages of sedentary, non-swarming *Schistocerca* species. Detailed information about taxon sampling is found in Supporting Information Table S1.

For all taxa, we generated nucleotide sequences through shotgun sequencing of genomic DNA using the Illumina platform. To extract high molecular weight DNA, we used Gentra Puregene Tissue Kit (Qiagen) following the manufacturer’s guidelines. The quality and concentration of DNA extracts were initially measured using either Qubit Fluorometer (Thermo Fisher Scientific) or DeNovix Spectrophotometer, and more thoroughly analysed using Fragment Analyzer. We used Nextera XT DNA Library Prep Kit for library preparation and performed 250 bp paired-end (PE) sequencing using MiSeq, 150 bp PE sequencing using NextSeq 500 or 125 bp PE sequencing using HiSeq 2500. Library preparation and next generation sequencing (NGS) were conducted at either Georgia Genomic Facility (MiSeq and NextSeq 500) or Texas A&M Genomics and Bioinformatics Service (HiSeq 2500). The resulting raw reads were quality-trimmed in CLC Genomics Workbench 8 (Qiagen). We used the MITObim pipeline^97^ to assemble mtgenomes *de novo* from the NGS reads. All newly assembled mtgenomes were first uploaded as raw fasta files to MITOS^98^ to identify open reading frames (ORFs) and tRNAs. The initial MITOS annotation was used as a guideline to delimit gene boundaries, and start and stop codons of each protein-coding gene were manually identified in Geneious 10.0.9 (Biomatters), following the recommendation by Cameron^99^. We also extracted histone 3 (H3) and histone 2B (H2B) genes from the shotgun sequence data by using ‘Map to Reference’ tool in Geneious. Using a grasshopper H3 sequence (KM853687) and a spider H2B sequence (XM_016058247) downloaded from GenBank as references, we used the Geneious mapper with low sensitivity to search for short reads that mapped to the reference sequences. This approach was very effective in extracting these two nuclear protein-coding genes from all 37 taxa. DNA sequence data generated for this study were deposited to GenBank and the accession numbers can be found in Supporting Information Table S1.

### Phylogenetic analyses

For both mitochondrial and nuclear protein-coding genes, we aligned based on the conservation of reading frames by first translating into amino acids and aligning individually in MUSCLE^100^ (Edgar, 2004) using default parameters in Geneious. Transfer RNA and ribosomal RNA genes were individually aligned in MAFFT^101^ using the E-INS-i algorithm also in Geneious. All these individual alignments were concatenated into a single matrix using SequenceMatrix^102^. We divided the data into a total of 69 data blocks (13 mitochondrial protein-coding genes, H3, and H2B divided into individual codon positions, 22 tRNAs, and 2 rRNAs) and used PartitionFinder v.2.1.1^103^ using the “greedy” algorithm (heuristic search) with branch lengths estimated as “unlinked” to search for the best-fit scheme as well as to estimate the model of nucleotide evolution for each partition. The final matrix consisted of 15,801 aligned bp and 37 taxa.

We performed both maximum likelihood (ML) and Bayesian analyses on the total evidence dataset. For the ML analysis, we used the best-fit partitioning scheme recommended by PartitionFinder with the GTRCAT model applied to each partition and analysed using RAxML 7.2.8^104^. Nodal support was evaluated using 1,000 replications of rapid bootstrapping implemented in RAxML. For the Bayesian analysis, we applied a different, unlinked model for each partition, as recommended by PartitionFinder, and ran four runs with four chains each for 100 million generations, sampling every 2500 generations in MrBayes 3.2.6^105^. We plotted the likelihood trace for each run to assess convergence in Tracer 1.6^106^, and discarded an average of 25% of each run as burn-in. Both analyses were run on XSEDE (Extreme Science and Engineering Discovery Environment, https://www.xsede.org) through the CIPRES Science Gateway^107^. The resulting trees were visualized in Geneious. Our aligned dataset and the resulting trees, as well as all associated data were deposited to Mendeley Data (http://dx.doi.org/10.17632/2g98nw9js5.1).

### Divergence time estimation

We estimated a time calibrated tree based on node dating in MrBayes with an independent gamma rate (IGR) as a relaxed clock model. We used an exponential prior (10) on the variance of the gamma distribution. We used a fossil taxon *Proschistocerca oligocaenica* Zeuner 1937 (37.2 to 33.9 Ma), which is the oldest definitive fossil of the subfamily Cyrtacanthacridinae^108^, to calibrate the root age, using an offset exponential distribution with minimal age 33.9 Ma and mean age 35.55 Ma. Although there is no fossil available for *Schistocerca*, we could infer the minimum age of at least one internal node based on a geological history. Three species (including *H. robustus*) are endemic to the Galapagos Islands and they form a clade, and it is generally accepted that colonisation events to the Galapagos have occurred over the last 3 to 4 million years^109^. So, we inferred that the common ancestor of these three species must have colonised the islands after some of the islands became colonisable. Thus, we used the ages of two oldest islands, South Plaza (4.2 Ma) and Espanola (3.2 Ma) to calibrate the node of this Galapagos clade (uniform, 3.2–4.2). We ran four runs with four chains each for 30 million generations, sampling every 2500 generations.

### Ancestral range estimation

We used the R package BioGeoBEARS^110^ in R^111^ to infer the biogeographical history of *Schistocerca* based on the time-calibrated tree. We defined eight areas to delimit geographical ranges indicating presence/absence of each species in each discrete area: Africa, South America, Central America, North America, Caribbean, Galapagos, Australia, and Indo-Pacific. We chose Caribbean and Galapagos as separate areas because of the endemic species in these areas and to emphasize the dispersal from South America to Galapagos. We included an adjacency matrix indicating connectivity between the discrete areas. The maximum number of ancestral areas was restricted to two in order to reflect the current distribution of the extant species. The analysis was non-time stratified because the age of the group was estimated to be geologically very recent with non-significant changes in the geographical areas.

### Ancestral character reconstruction

We compiled data on density-dependent phenotypic plasticity for all of the taxa included in this study (Supporting Information Table S2). Specifically, we examined two reaction norms that are commonly associated with locust phase polyphenism: colour and behaviour. We treated both reaction norms as binary traits. For both colour and behaviour, we coded as ‘non-plastic’ if there was a specific experimental study or observation showing the lack of any plastic reaction norm in a given species. For colour, we coded as ‘plastic’ if the species was known to show the development of black patterns or change in background colouration when crowded. For behaviour, we coded as ‘plastic’ for all locust species as well as some species that were experimentally shown to exhibit behavioural plasticity. For those species without clear data, we coded as unknown by giving 50% probability to both states. We estimated the ancestral character states for discrete traits using an MCMC based stochastic character mapping^71^ using phytools^112^ in R. Specifically, we simulated 500 stochastic character maps from our dataset to sample character histories from their posterior probability distribution. To test whether reaction norms in colour and behaviour have evolved in a correlated manner, we used Pagel’s character correlation test^67^, also using phytools in R.

## Electronic supplementary material

supplementary materials
