# Supplementary material for: Phylogeny of locusts and grasshoppers reveals complex evolution of density-dependent phenotypic plasticity
Source: Sci Rep. 2017 Jul 26;7:6606. doi: 10.1038/s41598-017-07105-y (PMC5529561; doi:10.1038/s41598-017-07105-y)
Supplement: Supplementary file 1 — supplementary materials [file 41598_2017_7105_MOESM1_ESM.pdf]

SUPPLEMENTARY INFORMATION

Phylogeny of locusts and grasshoppers reveals complex evolution of density-dependent phenotypic plasticity

Hojun Song[1]*, Bert Foquet[1], Ricardo Mariño-Pérez[1], Derek A. Woller[1]

[1]Department of Entomology, Texas A&M University, College Station, TX, U.S.A.

* Corresponding author: hsong@tamu.edu; 1-979-845-2481

Table S1. Taxon sampling, collecting information, and Genbank accession numbers. tRNA genes have not been submitted to Genbank due to their short sizes, but are available as alignments.

**Taxonomic Information**			
**TAMUIC-IGC Code**	**Genus**	**Species**	**Collecting Information**
OR1007	*Schistocerca*	*literosa*	Ecuador: Galapagos Is. Espanola, 2-V-1992, Coll. S Peck
OR1009	*Schistocerca*	*melanocera*	Ecuador: Galapagos Is. Isla Floreana, Finca Cruz fg. Of Cerro Communista, 28-III-1996, Coll. D Otte
OR1014	*Halmenus*	*robustus*	Ecuador: Galapagos Is. Isla Santa Cruz, trail to Tortuga Bay, 7-III-1996, Coll. D Otte
OR1080	*Schistocerca*	*obscura*	USA: FL, Suwannee Co. Suwannee River State Park, 30°23.064'N 83°09.655'W, 5-VIII-2002, Coll. H Song
OR1086	*Schistocerca*	*shoshone*	USA: UT, Utah Co., Goshen, 39°57'6"N 111°54'6"W, 13-IX-2008, Coll. H Song
OR1089	*Schistocerca*	*nitens (Costa Rica)*	Costa Rica: Limon Province, Veraqua Rainforest Research and Adventure Park, 9°56'20.76"N 83°12'39.96"W, 3-VII-2010, Coll. J Mugleston
OR1093	*Schistocerca*	*centralis*	Costa Rica, Puntarenas Province, Pacifico Central, Jaco Beach, 9°37'18.67"N 84°38'42.12"W, 29-VI-2010, Coll. J Mugleston
OR1122	*Schistocerca*	*lineata (Rubus)*	USA: TX, Colorado Co., Altair, 29°34.310'N and 96°27.135'W, 11-VII-2011, Coll. H Song, TJ Raszick, S Gotham
OR1158	*Schistocerca*	*serialis cubense*	USA: FL, Florida Keys, I-2013, Coll. DA Woller, S Gotham, R Mariño-Pérez
OR1161	*Schistocerca*	*pallens (Mexico)*	Mexico: San Luis Potosi, Rancho El Gualul Motorway, Estacion Manuel a Eban, 27-XI-2011, Coll. L Barrientos-Lozano
OR1166	*Schistocerca*	*gregaria*	Egypt, XI-2007, Coll. AA Haggag
OR1167	*Schistocerca*	*nitens (USA)*	USA: CA, San Diego, 10-XII-2007, Coll. A Latchininsky
OR1168	*Schistocerca*	*nitens (N. Mexico)*	Mexico: San Luis Potosi, Rancho El Gualul Motorway, Estacion Manuel a Eban, 27-XI-2011, Coll. L Barrientos-Lozano
OR1169	*Austracris*	*guttulosa*	Australia: Western Australia: 25 KM No Eneabba, 1-IX-2009, Coll. H Song
OR1170	*Cyrtacanthacris*	*aeruginosa*	South Africa: Western Cape Prov. Cederburg State Forest near Eikeboom, 7-II-2009, Coll. H Song
OR1171	*Acanthacris*	*ruficornis*	South Africa: Western Cape Prov. Theewaterskloof Dam, 10-II-2009, Coll. H Song
OR1173	*Nomadacris*	*septemfasciata*	Madagascar: Fianarantsoa Ranamafana National Park Centre ValBio, 19-22-XI-2008, Coll. G Svenson
OR1283	*Schistocerca*	*albolineata*	USA: AZ, Pima Co. Tucson Mts. Off Gates Pass Rd. 3-IX-2002, Coll. H.Song

**Taxonomic Information**			
**TAMUIC-IGC Code**	**Genus**	**Species**	**Collecting Information**
OR1284	*Schistocerca*	*alutacea*	USA: FL, Walton Co. DeFuniak Springs, off SR 90 W, Oakwood Hills, 30°45.418'N 86°16.661'W, 8-VIII-2002, Coll. H Song
OR1285	*Schistocerca*	*rubiginosa*	USA: FL Levy Co. off SR 121, 29°25.908'N 82°24.060'W, 6-VIII-2002, Coll. H Song
OR1287	*Schistocerca*	*ceratiola*	USA: FL, Putnam Co. Katherine Ordway Preserve on rosemary bush, 29°41.541'N 81°58.675'W, 7-VIII-2002, Coll. H Song
OR1288	*Schistocerca*	*lineata (Tamarix)*	USA: CO, Bent Co. John Martin Reservoir SWA, 15-VII-2004, Coll. H Song
OR1291	*Schistocerca*	*lineata (Ptelea)*	USA: TX, Burnet Co. Balcones Canyonlands National Wildlife Refuge, VII-2015. Coll. H Song
OR1294	*Schistocerca*	*quisqueya*	Dominican Republic: L RD-060, Monte Rio, 17-XI-2002, Coll. D Perez-Gelabert
OR1295	*Schistocerca*	*flavofasciata*	British Virgin Island: Guana I. 9-X-2002, Coll. B Valentine
OR1296	*Schistocerca*	*pallens (Brazil)*	Brazil: Piaui, Baixada Das Mulheres, Sao Raimundo Nonato, VIII-2005, Coll. S Bybee, K Dittmar
OR1297	*Schistocerca*	*piceifrons*	Mexico: San Luis Potosi, Rancho El Gualul Motorway, Estacion Manuel a Eban, 27-XI-2011, Coll. L Barrientos-Lozano
OR1300	*Schistocerca*	*caribbeana*	USVI: St. John, Yawzi Point Trail, 10-VII-2013, Coll. H Song
OR1302	*Schistocerca*	*cohni*	Mexico: Oaxaca, Hwy#190 Oaxaca Tehuantepec, 9-XII-2011, Coll. P Fontana, P Tirello, DA Woller, R Mariño-Pérez
OR1307	*Schistocerca*	*americana*	USA: FL, Levy Co. Off SR121, 6-VIII-2002, Coll. H Song
OR1379	*Schistocerca*	*damnifica*	USA: FL, Levy Co. Off SR121, 6-VIII-2002, Coll. H Song
OR1380	*Schistocerca*	*socorro*	Mexico: Islas Revillagigedo, Isla Socorro, 20-X-2004,Coll. H Song
OR1381	*Valanga*	*sp*	PNG: Eastern Highlands Province, Herowana Village Crater Mountain Research Area, 15-19-VII-2001, Coll. S Bradler, KJ Jarvis, G Svenson
OR1642	*Schistocerca*	*cancellata*	Argentina: Catamarca, I-2016, Coll. M Pocco
OR1665	*Schistocerca*	*nitens (S. Mexico)*	Mexico: Michoacan, Carapan km #113 N19°50'57.5" W102°03'27.8", 05-XII-2013, Coll. R Mariño-Pérez, P Fontana, O Sanabria-Urban
OR184	*Anacridium*	*incisum*	Namibia: Etosha NP Devilwater Spring (18 59 266 S 15 15 593 E), 18-V-2004, Coll. KB Miller
OR185	*Schistocerca*	*pallens (Bolivia)*	Bolivia: Santa Cruz Co. Conception (S 16 26.790 W 062 28.984), 12-I-2004, Coll. G Svenson, SL Cameron, S Bybee

TAMUIC-IGC Code	Genus	Species	ATP6	ATP8	COI	COII	COIII	CYTB
OR1007	*Schistocerca*	*literosa*	KY980825	N/A	KY980892	KY980929	KY980966	KY981002
OR1009	*Schistocerca*	*melanocera*	KY980826	KY980859	KY980893	KY980930	KY980967	KY981003
OR1014	*Halmenus*	*robustus*	KY980827	KY980860	KY980894	KY980931	KY980968	KY981004
OR1080	*Schistocerca*	*obscura*	KY980828	KY980861	KY980895	KY980932	KY980969	KY981005
OR1086	*Schistocerca*	*shoshone*	KY980829	KY980862	KY980896	KY980933	KY980970	KY981006
OR1089	*Schistocerca*	*nitens (Costa Rica)*	KY980830	KY980863	KY980897	KY980934	KY980971	KY981007
OR1093	*Schistocerca*	*centralis*	KY980831	KY980864	KY980898	KY980935	KY980972	KY981008
OR1122	*Schistocerca*	*lineata (Rubus)*	KY980832	KY980865	KY980899	KY980936	KY980973	KY981009
OR1158	*Schistocerca*	*serialis cubense*	KY980833	KY980866	KY980900	KY980937	KY980974	KY981010
OR1161	*Schistocerca*	*pallens (Mexico)*	KY980834	KY980867	KY980901	KY980938	KY980975	KY981011
OR1166	*Schistocerca*	*gregaria*	KY980835	KY980868	KY980902	KY980939	KY980976	KY981012
OR1167	*Schistocerca*	*nitens (USA)*	KY980836	KY980869	KY980903	KY980940	KY980977	KY981013
OR1168	*Schistocerca*	*nitens (N. Mexico)*	KY980837	KY980870	KY980904	KY980941	KY980978	KY981014
OR1169	*Austracris*	*guttulosa*	KY980838	KY980871	KY980905	KY980942	KY980979	KY981015
OR1170	*Cyrtacanthacris*	*aeruginosa*	KY980839	KY980872	KY980906	KY980943	KY980980	KY981016
OR1171	*Acanthacris*	*ruficornis*	KY980840	KY980873	KY980907	KY980944	KY980981	KY981017
OR1173	*Nomadacris*	*septemfasciata*	KY980841	KY980874	KY980908	KY980945	KY980982	KY981018
OR1283	*Schistocerca*	*albolineata*	KY980842	KY980875	KY980909	KY980946	KY980983	KY981019
OR1284	*Schistocerca*	*alutacea*	KY980843	KY980876	KY980910	KY980947	KY980984	KY981020
OR1285	*Schistocerca*	*rubiginosa*	KY980844	KY980877	KY980911	KY980948	KY980985	KY981021
OR1287	*Schistocerca*	*ceratiola*	KY980845	KY980878	KY980912	KY980949	KY980986	KY981022
OR1288	*Schistocerca*	*lineata (Tamarix)*	KY980846	KY980879	KY980913	KY980950	KY980987	KY981023
OR1291	*Schistocerca*	*lineata (Ptelea)*	KY980847	KY980880	KY980914	KY980951	KY980988	KY981024
OR1294	*Schistocerca*	*quisqueya*	KY980848	KY980881	KY980915	KY980952	KY980989	KY981025
OR1295	*Schistocerca*	*flavofasciata*	N/A	N/A	KY980916	KY980953	N/A	N/A
OR1296	*Schistocerca*	*pallens (Brazil)*	KY980849	KY980882	KY980917	KY980954	KY980990	KY981026
OR1297	*Schistocerca*	*piceifrons*	KY980850	KY980883	KY980918	KY980955	KY980991	KY981027
OR1300	*Schistocerca*	*caribbeana*	KY980851	KY980884	KY980919	KY980956	KY980992	KY981028
OR1302	*Schistocerca*	*cohni*	KY980852	KY980885	KY980920	KY980957	KY980993	KY981029
OR1307	*Schistocerca*	*americana*	KY980853	KY980886	KY980921	KY980958	KY980994	KY981030
OR1379	*Schistocerca*	*damnifica*	KY980854	N/A	KY980922	KY980959	KY980995	KY981031
OR1380	*Schistocerca*	*socorro*	KY980855	KY980887	KY980923	KY980960	KY980996	KY981032
OR1381	*Valanga*	*sp*	N/A	N/A	KY980924	KY980961	KY980997	N/A
OR1642	*Schistocerca*	*cancellata*	KY980856	KY980888	KY980925	KY980962	KY980998	KY981033
OR1665	*Schistocerca*	*nitens (S. Mexico)*	KY980857	KY980889	KY980926	KY980963	KY980999	KY981034
OR184	*Anacridium*	*incisum*	KY980823	KY980858	KY980890	KY980927	KY980964	KY981000
OR185	*Schistocerca*	*pallens (Bolivia)*	KY980824	N/A	KY980891	KY980928	KY980965	KY981001

TAMUIC-IGC Code	Genus	Species	ND1	ND2	ND3	ND4	ND4L	ND5
OR1007	*Schistocerca*	*literosa*	KY981037	KY981072	KY981109	KY981144	KY981180	N/A
OR1009	*Schistocerca*	*melanocera*	KY981038	KY981073	KY981110	KY981145	KY981181	KY981211
OR1014	*Halmenus*	*robustus*	KY981039	KY981074	KY981111	KY981146	KY981182	KY981212
OR1080	*Schistocerca*	*obscura*	KY981040	KY981075	KY981112	KY981147	N/A	KY981213
OR1086	*Schistocerca*	*shoshone*	KY981041	KY981076	KY981113	KY981148	N/A	KY981214
OR1089	*Schistocerca*	*nitens (Costa Rica)*	KY981042	KY981077	KY981114	KY981149	KY981183	N/A
OR1093	*Schistocerca*	*centralis*	KY981043	KY981078	KY981115	KY981150	KY981184	KY981215
OR1122	*Schistocerca*	*lineata (Rubus)*	KY981044	KY981079	KY981116	KY981151	KY981185	KY981216
OR1158	*Schistocerca*	*serialis cubense*	KY981045	KY981080	KY981117	KY981152	KY981186	KY981217
OR1161	*Schistocerca*	*pallens (Mexico)*	KY981046	KY981081	KY981118	KY981153	KY981187	KY981218
OR1166	*Schistocerca*	*gregaria*	KY981047	KY981082	KY981119	KY981154	KY981188	KY981219
OR1167	*Schistocerca*	*nitens (USA)*	KY981048	KY981083	KY981120	KY981155	KY981189	KY981220
OR1168	*Schistocerca*	*nitens (N. Mexico)*	KY981049	KY981084	KY981121	KY981156	KY981190	KY981221
OR1169	*Austracris*	*guttulosa*	KY981050	KY981085	KY981122	KY981157	KY981191	KY981222
OR1170	*Cyrtacanthacris*	*aeruginosa*	KY981051	KY981086	KY981123	KY981158	KY981192	KY981223
OR1171	*Acanthacris*	*ruficornis*	KY981052	KY981087	KY981124	KY981159	KY981193	KY981224
OR1173	*Nomadacris*	*septemfasciata*	KY981053	KY981088	KY981125	KY981160	KY981194	KY981225
OR1283	*Schistocerca*	*albolineata*	KY981054	KY981089	KY981126	KY981161	KY981195	KY981226
OR1284	*Schistocerca*	*alutacea*	KY981055	KY981090	KY981127	KY981162	KY981196	KY981227
OR1285	*Schistocerca*	*rubiginosa*	KY981056	KY981091	KY981128	KY981163	KY981197	KY981228
OR1287	*Schistocerca*	*ceratiola*	KY981057	KY981092	KY981129	KY981164	KY981198	KY981229
OR1288	*Schistocerca*	*lineata (Tamarix)*	KY981058	KY981093	KY981130	KY981165	KY981199	KY981230
OR1291	*Schistocerca*	*lineata (Ptelea)*	KY981059	KY981094	KY981131	KY981166	KY981200	KY981231
OR1294	*Schistocerca*	*quisqueya*	KY981060	KY981095	KY981132	KY981167	KY981201	KY981232
OR1295	*Schistocerca*	*flavofasciata*	N/A	KY981096	N/A	N/A	N/A	N/A
OR1296	*Schistocerca*	*pallens (Brazil)*	KY981061	KY981097	KY981133	KY981168	KY981202	KY981233
OR1297	*Schistocerca*	*piceifrons*	KY981062	KY981098	KY981134	KY981169	KY981203	KY981234
OR1300	*Schistocerca*	*caribbeana*	KY981063	KY981099	KY981135	KY981170	KY981204	KY981235
OR1302	*Schistocerca*	*cohni*	KY981064	KY981100	KY981136	KY981171	KY981205	KY981236
OR1307	*Schistocerca*	*americana*	KY981065	KY981101	KY981137	KY981172	KY981206	KY981237
OR1379	*Schistocerca*	*damnifica*	KY981066	KY981102	KY981138	KY981173	N/A	KY981238
OR1380	*Schistocerca*	*socorro*	KY981067	KY981103	KY981139	KY981174	KY981207	KY981239
OR1381	*Valanga*	*sp*	N/A	KY981104	KY981140	KY981175	N/A	KY981240
OR1642	*Schistocerca*	*cancellata*	KY981068	KY981105	KY981141	KY981176	KY981208	KY981241
OR1665	*Schistocerca*	*nitens (S. Mexico)*	KY981069	KY981106	KY981142	KY981177	KY981209	KY981242
OR184	*Anacridium*	*incisum*	KY981035	KY981070	KY981107	KY981143	KY981178	KY981210
OR185	*Schistocerca*	*pallens (Bolivia)*	KY981036	KY981071	KY981108	KY981144	KY981179	N/A

TAMUIC-IGC Code	Genus	Species	ND6	12S	16S	H3	H2B
OR1007	*Schistocerca*	*literosa*	KY981245	KY980753	KY980789	KY923759	KY923707
OR1009	*Schistocerca*	*melanocera*	KY981246	KY980754	KY980790	KY923760	KY923708
OR1014	*Halmenus*	*robustus*	KY981247	KY980755	KY980791	KY923771	KY923709
OR1080	*Schistocerca*	*obscura*	N/A	KY980756	KY980792	KY923763	KY923710
OR1086	*Schistocerca*	*shoshone*	N/A	KY980757	KY980793	KY923769	KY923711
OR1089	*Schistocerca*	*nitens (Costa Rica)*	N/A	KY980758	KY980794	KY923778	KY923712
OR1093	*Schistocerca*	*centralis*	KY981248	KY980759	KY980795	KY923761	KY923713
OR1122	*Schistocerca*	*lineata (Rubus)*	N/A	KY980760	KY980796	KY923770	KY923714
OR1158	*Schistocerca*	*serialis cubense*	KY981249	KY980761	KY980797	KY923772	KY923715
OR1161	*Schistocerca*	*pallens (Mexico)*	KY981250	KY980762	KY980798	KY923755	KY923716
OR1166	*Schistocerca*	*gregaria*	KY981251	KY980763	KY980799	KY923753	KY923717
OR1167	*Schistocerca*	*nitens (USA)*	KY981252	KY980764	KY980800	KY923758	KY923718
OR1168	*Schistocerca*	*nitens (N. Mexico)*	KY981253	KY980765	KY980801	KY923777	KY923719
OR1169	*Austracris*	*guttulosa*	KY981254	KY980766	KY980802	KY923752	KY923720
OR1170	*Cyrtacanthacris*	*aeruginosa*	KY981255	KY980767	N/A	KY923742	KY923721
OR1171	*Acanthacris*	*ruficornis*	N/A	KY980768	KY980803	KY923743	KY923741
OR1173	*Nomadacris*	*septemfasciata*	KY981256	KY980769	KY980804	KY923749	KY923722
OR1283	*Schistocerca*	*albolineata*	N/A	KY980770	KY980805	KY923766	KY923723
OR1284	*Schistocerca*	*alutacea*	KY981257	KY980771	KY980806	KY923764	KY923724
OR1285	*Schistocerca*	*rubiginosa*	KY981258	KY980772	KY980807	KY923776	KY923725
OR1287	*Schistocerca*	*ceratiola*	KY981259	KY980773	KY980808	KY923767	KY923726
OR1288	*Schistocerca*	*lineata (Tamarix)*	KY981260	KY980774	KY980809	KY923768	KY923727
OR1291	*Schistocerca*	*lineata (Ptelea)*	KY981261	KY980775	KY980810	KY923765	KY923728
OR1294	*Schistocerca*	*quisqueya*	KY981262	KY980776	KY980811	KY923750	KY923729
OR1295	*Schistocerca*	*flavofasciata*	N/A	KY980777	KY980812	KY923746	KY923730
OR1296	*Schistocerca*	*pallens (Brazil)*	KY981263	KY980778	KY980813	KY923756	KY923731
OR1297	*Schistocerca*	*piceifrons*	KY981264	KY980779	KY980814	KY923774	KY923732
OR1300	*Schistocerca*	*caribbeana*	KY981265	KY980780	KY980815	KY923747	KY923733
OR1302	*Schistocerca*	*cohni*	KY981266	KY980781	KY980816	KY923757	KY923734
OR1307	*Schistocerca*	*americana*	KY981267	KY980782	KY980817	KY923773	KY923735
OR1379	*Schistocerca*	*damnifica*	N/A	KY980783	KY980818	KY923762	KY923736
OR1380	*Schistocerca*	*socorro*	KY981268	KY980784	KY980819	KY923775	KY923737
OR1381	*Valanga*	*sp*	N/A	N/A	KY980820	KY923744	KY923738
OR1642	*Schistocerca*	*cancellata*	KY981269	KY980785	KY980821	KY923748	KY923739
OR1665	*Schistocerca*	*nitens (S. Mexico)*	KY981270	KY980786	KY980822	KY923751	KY923740
OR184	*Anacridium*	*incisum*	KY981243	KY980751	KY980787	KY923745	KY923705
OR185	*Schistocerca*	*pallens (Bolivia)*	KY981244	KY980752	KY980788	KY923754	KY923706

Table S2. Survey of density-dependent phenotypic plasticity of the species included in this study.

Species	Reaction norm in colour	Reaction norm in behaviour	Data Source
Anacridium incisum	unknown	unknown	
Acanthacris ruficornis	plastic	non-plastic	Rowell (1971); COPR (1982); Popov (1989); Song and Wenzel (2008)
Cyrtacanthacris aeruginosa	unknown	non-plastic	COPR (1982); Song and Wenzel (2008)
Nomadacris septemfasciata	plastic	plastic	Dean (1967); Popov (1989); Song and Wenzel (2008)
Valanga sp.	unknown	unknown	
Austracris guttulosa	plastic	plastic	Elder (1989, 1991, 1997); Song and Wenzel (2008)
Halmenus robustus	unknown	unknown	
Schistocerca albolineata	unknown	non-plastic	Song (2004)
Schistocerca alutacea	unknown	non-plastic	Song (2004)
Schistocerca americana	plastic	plastic	Gotham and Song (2013)
Schistocerca cancellata	plastic	plastic	Waloff and Pedgley (1986); Song and Wenzel (2008)
Schistocerca caribbeana	non-plastic	non-plastic	Song, unpublished
Schistocerca centralis	unknown	unknown	
Schistocerca ceratiola	non-plastic	non-plastic	Song, unpublished
Schistocerca cohni	unknown	unknown	
Schistocerca damnifica	plastic	non-plastic	Song, unpublished
Schistocerca flavofasciata	plastic	non-plastic	Kevan (1943)
Schistocerca gregaria	plastic	plastic	Uvarov (1966); Roessingh et al. (1993); Song and Wenzel (2008)
Schistocerca lineata (*Ptelea*)	plastic	non-plastic	Sword (1999); Song and Wenzel (2008)
Schistocerca lineata (*Rubus*)	plastic	non-plastic	Sword (1999); Song and Wenzel (2008)
Schistocerca lineata (*Tamarix*)	plastic	non-plastic	Raszick and Song (2016)
Schistocerca literosa	unknown	unknown	
Schistocerca melanocera	unknown	unknown	
Schistocerca nitens (Costa Rica)	unknown	unknown	
Schistocerca nitens (N. Mexico)	unknown	unknown	
Schistocerca nitens (S. Mexico)	unknown	unknown	
Schistocerca nitens (USA)	non-plastic	non-plastic	Rowell and Cannis (1971); Song and Wenzel (2008)
Schistocerca obscura	plastic	non-plastic	Duck (1944); Song and Wenzel (2008)
Schistocerca pallens (Bolivia)	plastic	non-plastic	Antoniou and Robinson (1974); Song and Wenzel (2008)
Schistocerca pallens (Brazil)	plastic	non-plastic	Antoniou and Robinson (1974); Song and Wenzel (2008)

Species	Reaction norm in colour	Reaction norm in behaviour	Data Source
Schistocerca pallens (Mexico)	plastic	non-plastic	Antoniou and Robinson (1974); Song and Wenzel (2008)
Schistocerca piceifrons	plastic	plastic	Harvey (1983); Song and Wenzel (2008)
Schistocerca quisqueya	unknown	unknown	
Schistocerca rubiginosa	plastic	non-plastic	Song, unpublished
Schistocerca serialis cubense	plastic	plastic	Gotham and Song (2013)
Schistocerca shoshone	unknown	non-plastic	Song (2004)
Schistocerca socorro	unknown	unknown	

References

1. Antoniou, A. & Robinson, C. J. Laboratory studies on the effect of crowding on phase and the life history of *Schistocerca pallens* (Thunberg) (Orthoptera: Acrididae: Cyrtacanthacridinae). *J. Nat. Hist.* **8**, 701-715 (1974).
2. COPR. *The locust and grasshopper agricultural manual*. (Centre for Overseas Pest Research, 1982).
3. Dean, G. J. W. Observations on the structure of hopper bands and movement of hoppers of the red locust (*Nomadacris septemfasciata* Serville). *Journal of the Entomological Society of Southern Africa* **30**, 1-17 (1967).
4. Duck, L. G. The bionomics of *Schistocerca obscura* (Fabr). *J. Kans. Entomol. Soc.* **17**, 105-119 (1944).
5. Elder, R. J. Laboratory studies on the life history of *Nomadacris guttulosa* (Walker) (Orthoptera: Acrididae). *J. Aust. Entomol. Soc.* **28**, 247-253 (1989).
6. Elder, R. J. Laboratory studies of environmental factors affecting sexual maturation in *Nomadacris guttulosa* (Walker) (Orthoptera: Acrididae). *J. Aust. Entomol. Soc.* **30**, 169-181 (1991).
7. Elder, R. J. Bionomics of *Austracris guttulosa* (Walker) (Orthoptera: Acrididae) during the 1970-75 oubreak in Queensland, Australia. *Aust. J. Entomol.* **36**, 57-67 (1997).
8. Gotham, S. & Song, H. Non-swarming grasshoppers exhibit density-dependent phenotypic plasticity reminiscent of swarming locusts. *J. Insect Physiol.* **59**, 1151-1159 (2013).
9. Harvey, A. W. *Schistocerca piceifrons* (Walker) (Orthoptera: Acrididae), the swarming locust of tropical America: a review. *Bull. ent. Res.* **73**, 171-184 (1983).
10. Kevan, D. K. M. An account of *Schistocerca flavofasciata* (De Geer 1773) in Trinidad (Orthoptera: Acrididae). *Bull. Entomol. Res.* **34**, 291-310 (1943).
11. Popov, G. B. *Nymphs of the Sahelian grasshoppers: an illustrated guide*. (Overseas Development Natural Resources Institute, 1989).
12. Raszick, T. J. & Song, H. The ecotype paradigm: testing the concept in an ecologically divergent grasshopper. *Insect Syst. Evol.* **47**, 363-387 (2016).
13. Roessingh, P., Simpson, S. J. & James, S. Analysis of phase-related changes in behaviour of desert locust nymphs. *Proc. R. Soc. Lond. B* **252**, 43-49 (1993).
14. Rowell, C. H. F. The variable coloration of the acridoid grasshoppers. *Adv. Insect Physiol.* **8**, 145-198 (1971).
15. Rowell, C. H. F. & Cannis, T. L. Environmental factors affecting the green/brown polymorphism in the cyrtacanthacridine grasshopper *Schistocerca vaga* (Scudder). *Acrida* **1**, 69-77 (1971).
16. Song, H. Revision of the Alutacea Group of genus *Schistocerca* (Orthoptera: Acrididae: Cyrtacanthacridinae). *Ann. Entomol. Soc. Am.* **97**, 420-436 (2004).
17. Song, H. & Wenzel, J. W. Phylogeny of bird-grasshopper subfamily Cyrtacanthacridinae (Orthoptera: Acrididae) and the evolution of locust phase polyphenism. *Cladistics* **24**, 515-542 (2008).
18. Sword, G. A. Density-dependent warning coloration. *Nature* **397**, 217 (1999).
19. Uvarov, B. P. *Grasshoppers and Locusts, Vol. 1*. (Cambridge University Press, 1966).
20. Waloff, Z. & Pedgley, D. E. Comparative biogeography and biology of the South American locust, *Schistocerca cancellata* (Serville), and the South African desert locust, *S. gregaria flaviventris* (Burmeister) (Orthoptera: Aciriididae): a review. *Bull. ent. Res.* **76**, 1-20 (1986).
